# American Medical College Association

**Published:** 1882-07

**Authors:** 


THE AMERICAN MEDICAL COLLEGE ASSOCIATION.

This association, or what there is left of it, held a meeting in Cincinnati recently, the proceedings of which are published in a recent number of the Detroit *Lancet.* We notice that a list of colleges is given as having withdrawn from the association since it adopted the requirement of three years' study and three consecutive annual courses of college instruction, in such a way as to convey the impression to the reader that all the colleges mentioned withdrew *because* of the *adoption* of such requirement. Yet the Chicago Medical College is included in the list, when the officers of the association well knew its withdrawal was based exclusively on the fact that if the association ever did anything it would recede from such requirement, and the College was not willing to accompany it in any such retrograde movement.

---

The *British Medical Journal* says that "the commission appointed by the French Academy of Sciences to investigate the question of the advisability of modifying the system of quarantine in Egypt have sent in their report." But it is no longer a question of quarantine, but one of exclusion.

